# Unveiling the hidden story of anemia among Indian Muslim women: a comprehensive analysis from 1998 to 2021

**DOI:** 10.3389/fnut.2025.1592436

**Published:** 2025-06-30

**Authors:** Zeenat Hashmi, Ashish Singh

**Affiliations:** SJM School of Management, Indian Institute of Technology Bombay, Mumbai, India

**Keywords:** anemia, health disparities, inequality, iron deficiency, Muslim women, women’s health

## Abstract

**Background:**

Iron deficiency among women of reproductive age, driven by factors such as inadequate dietary intake, menstrual blood loss, and heightened iron demands during pregnancy, remains a global public health concern. This research focuses on the prevalence of anemia among Muslim women in India over the last two decades, with a particular focus on disparities and trends.

**Methods:**

Anemia among Muslim women of reproductive age (15–49 years) is studied using the four rounds of the National Family Health Survey (NFHS-2 to NFHS-5) to analyze the trends and disparities using bivariate cross-tabulations, concentration index, P/R ratios, and odds ratios across different socioeconomic factors. This study analyzes data from 212,837 Muslim women of reproductive age, collected through four rounds of the National Family Health Survey between 1998 and 2021.

**Results:**

The findings reveal a concerning upward trend from 48.77 to 55.6% (1998–2021) in anemia prevalence among Muslim women in India. Anemia is consistently found to be highest in the age group of 15–19 across all the surveys and reported to be 59.14% in NFHS-5. Geographically, the Northeastern and Eastern regions exhibit the highest anemia prevalence rates, at 72.12 and 60.5% in 1998–99, respectively, which decreased to 41.41 and 55.95% in 2015–16, but again rose by 17.74 and 8.72% in 2019–21, respectively. The Western region increased from 37.6% in 1998–99 to 51.76% in 2019–21. Furthermore, rural areas witness a strikingly higher anemia prevalence among women, exceeding urban areas by over 8%. The Scheduled Caste/Scheduled Tribe (SC/ST) populations consistently bear the highest anemia burden. Economic disparities are evident, as wealth quintiles and education attainment display a transparent gradient, with the poorest quintile and no education consistently having the highest odds of anemia.

**Conclusion:**

The socially disadvantaged groups, economically backward and less educated women, have constantly shown the highest prevalence of anemia for the period of the past two decades. The policies to improve public health should specifically focus on the most vulnerable sections of society. There is a need to modify existing public policies and improve population health in the context of the most vulnerable sections in developing countries.

## 1 Introduction

Anemia occurs when the blood lacks enough healthy red blood cells or hemoglobin, essential for oxygen transport. This leads to symptoms like fatigue, weakness, dizziness, and shortness of breath (1). The most common cause is iron deficiency, which hampers hemoglobin production. However, other micronutrient deficiencies, such as folate, vitamin B12, zinc, and vitamin C are also significant contributors to anemia, particularly in low-resource settings like India (2–4). Adolescents, particularly girls from low-income households, tend to consume less diverse diets and are thus more vulnerable to micronutrient deficiencies (5). These patterns call for improving dietary intake as well as nutrient bioavailability, especially for iron and folate. In this context, anemia must be understood not only as a nutritional issue but also as a socio-structural problem shaped by poverty, access, gender inequity, and other socioeconomic determinants (6, 7).

Women of reproductive age (WRA) are especially vulnerable due to factors such as heavy menstruation, frequent pregnancies, and increased nutritional requirements (8, 9). Anemia affects nearly a quarter of the global population, with women and children being the most vulnerable groups (10). Anemia’s impacts extend beyond its physiological symptoms, manifesting as a multifaceted health, societal, and economic issue (11). Among children, anemia is associated with impaired cognitive and motor development, delayed academic progress, and limited social interactions, which can hinder their future economic productivity (12). Among women, anemia contributes to maternal morbidity and is linked to adverse pregnancy outcomes such as preterm birth, low birth weight, and stillbirth, as well as increased maternal mortality (9, 13). Furthermore, maternal anemia has intergenerational effects, leading to poor health outcomes in children, including stunting and impaired cognitive development (14). Nutritional and perinatal deficiencies have been found to be leading contributors to increased morbidity and mortality among women of reproductive age in India (15). Despite policy efforts that historically focused on iron and folic acid supplementation, recent studies emphasize the need to address multiple micronutrient deficiencies and structural determinants of anemia in tandem (7, 16). Studies also indicate that the relation between dietary iron intake and anemia is modulated by coexisting nutritional deficiencies and non-nutritional factors such as poverty, hemoglobinopathies, and infections (17–19). Beyond its health implications, anemia imposes significant economic burdens (20). The productivity losses associated with anemia stem from reduced physical capacity, fatigue, and impaired cognitive function. Investments in addressing anemia are therefore economically beneficial, with every US$ 1 invested in anemia reduction among women projected to yield US$ 12 in economic returns (21). These compounded impacts on health and productivity establish anemia as not just a medical condition but also a significant developmental and social justice challenge (20, 22).

Long-term anemia can even impact productivity and overall quality of life (12). To address anemia, strategies such as improving diets, iron and folic acid supplementation, and treating underlying causes like infections are crucial. The WHO has set a global target to reduce anemia in women of reproductive age by 50% by 2025 (1). Globally, approximately 24.8% of the population is affected by anemia (23). Among women of reproductive age, the prevalence in 2019 was 29.9%, equating to over half a billion women (24). Non-pregnant women experienced a global anemia prevalence of 30.2%, while 41.8% of pregnant women were affected in the same period (25).

India has one of the highest burdens of anemia among women of reproductive age, with over 52.2% of pregnant women and 59.1% of women aged 15–49 affected, as per the National Family Health Survey (26). The persistent burden highlights the need for a deeper understanding of its underlying determinants. India has implemented various programs to combat anemia, such as the Anemia Mukt Bharat initiative launched in 2018, the Public Distribution System (PDS), and the Integrated Child Development Services (ICDS) program, which also focuses on providing iron and folic acid supplementation, deworming, and dietary counseling, particularly for women and adolescent girls (27, 28). However, despite these efforts, progress has been slow, primarily due to the lack of effective implementation and socioeconomic barriers, and has failed to reach the most marginalized communities, such as SCs and STs (29, 30). Evidence suggests that combining nutritional interventions with socioeconomic empowerment programs, such as skill development and education initiatives, can yield better outcomes in reducing anemia (8).

Over the past two decades, data from the National Family Health Surveys (NFHS-2, 1998–99; NFHS-3, 2005–06; NFHS-4, 2015–16; and NFHS-5, 2019–21) show persistent anemia disparities across socioeconomic groups increasing from 48.7% in NFHS-2 (1998–99) to 55.6% in NFHS-5 (31, 32). The NFHS is a nationally representative stratified survey, including 90,303 women in NFHS-2, 124,385 women in NFHS-3, 699,686 women in NFHS-4, and 724,115 women of reproductive age (16–49 years) in NFHS-5. Among these, Muslim women of reproductive age accounted for 11.93, 13.45, 13.52, and 12.52%, respectively. The high prevalence of anemia in India is deeply linked to its socioeconomic and cultural structures, where caste, class, gender, ethnicity, and religion shape access to healthcare, nutrition, and education (27). Existing research on anemia in India has primarily focused on single-axis frameworks examining gender, caste, or class independently. For instance, studies have highlighted the association of anemia with caste (33, 34), ethnicity (6, 35), and gender (27, 36, 37). Similarly, research has shown how socioeconomic factors such as low educational status (38), poor housing conditions (14), and hygiene practices (39) influence anemia prevalence. Studies show that the Northeastern and Eastern states consistently report the highest prevalence rates, with over 60% of women affected in some states like Bihar and Assam (40). Rural areas exhibit a higher burden compared to urban areas, as limited healthcare access, poor sanitation, and dietary inadequacies are more common in rural regions (24). Women from Scheduled Caste (SC) and Scheduled Tribe (ST) communities experience higher rates of anemia due to systemic marginalization, poor living conditions, and limited healthcare access (33, 41, 42). Wealth quintiles and educational attainment display a clear gradient, with the poorest and least educated women being the most vulnerable (14). However, these studies fail to account for how multiple socioeconomic dimensions interact to shape health outcomes, leaving critical gaps in understanding the lived realities of marginalized groups such as Muslim women. They experience compounded disadvantages due to socioeconomic marginalization and gender-based discrimination, as highlighted by the Sachar Committee, 2006 (43). However, their health challenges remain underrepresented in research.

Intersectionality, introduced by Crenshaw (1989), helps explain how overlapping social identities such as caste, class, gender, and religion, shape anemia risk. A rural Muslim woman, for example, may face disadvantages from poverty, gender discrimination, and religious marginalization, intensifying her vulnerability (44). Despite its relevance, intersectionality is underutilized in Indian health research, which often adopts single-axis approaches, overlooking the interplay of multiple disadvantages (45, 46).

This study seeks to fill these gaps by adopting an intersectional framework to examine the prevalence and determinants of anemia among Muslim women in India. It explores how socioeconomic factors such as caste, class, age, place of residence, education, and geographical region interact to influence anemia prevalence among Muslim women. This research also aligns with global health goals, such as the United Nations’ 2030 Agenda for Sustainable Development and the World Health Assembly’s Comprehensive Implementation Plan on Maternal, Infant, and Young Child Nutrition. By focusing on Muslim women, a group often overlooked in public health policy, this study contributes to the broader effort to achieve health equity and ensure “health for all.” This research addresses a critical gap in the literature by applying an intersectional lens to anemia prevalence among Muslim women in India. By shedding light on the unique challenges faced by Muslim women, this study underscores the importance of inclusive and equity-driven public health strategies and tests the effectiveness of current policies with respect to a specific section of society.

## 2 Materials and methods

This study utilizes data from the four rounds of the National Family Health Surveys (NFHS) conducted in India, spanning over two decades: NFHS-2 (1998–99), NFHS-3 (2005–06), NFHS-4 (2015–16), and NFHS-5 (2019–21). The NFHS surveys, implemented by the Ministry of Health and Family Welfare (MoHFW) with technical support from the International Institute for Population Sciences (IIPS), provide nationally representative data on various health and demographic indicators. These surveys are instrumental in examining the prevalence and trends of anemia among different population groups, including Muslim women. The study focuses on women of reproductive age (15–49 years), specifically analyzing the subset of Muslim women within this demographic. The sample size for Muslim women varies across the NFHS rounds: NFHS-2 includes 10,775 ever-married Muslim women, NFHS-3 expands to 16,742 Muslim women (including both married and unmarried individuals), NFHS-4 captures 94,591 Muslim women, and NFHS-5 incorporates 90,729 Muslim women. The total pooled sample size of Muslim women across the four NFHS rounds used in this study is 212,837.

This study aims to examine the trends and disparities in the prevalence of any and severe anemia among Muslim women among Muslim women of reproductive age (15–49 years) across different socioeconomic and demographic factors such as caste (Scheduled Caste, Scheduled Tribe, Other Backward Classes, General), age groups (e.g., 15–19, 20–29, 30–39, 40–49), place of residence (rural and urban), Education levels (no education, primary, secondary, higher), wealth quintiles (poorest, poorer, middle, richer, richest), regions (e.g., North, South, East, West, Northeast). Anemia is measured using hemoglobin levels among women in India and worldwide. According to the WHO cutoffs, Anemia is typically defined as a condition where the hemoglobin levels in a woman’s blood fall below a certain threshold, which is 12 g/dL (any anemia), 10.00–11.90 g/dL (mild), 7.00–9.90 (moderate) g/dL, and <7.00 (severe) g/dL (47). In this study, “severe” (hemoglobin <7.00 g/dL) and “any” (hemoglobin <12.00 g/dL) categories are used for the analysis to make the analysis comprehensible and inclusive. To maintain consistency and transparency, the diagnosis of anemia in this study is based solely on hemoglobin concentration, following World Health Organization (WHO) thresholds. Women were categorized as anemic if their hemoglobin level was below 12.0 g/dL (for non-pregnant women) or below 11.0 g/dL (for pregnant women). Observations were excluded if they had missing hemoglobin values, biologically implausible or flagged hemoglobin readings, or incomplete pregnancy-related information that made the application of the relevant hemoglobin cutoff unfeasible. These exclusions ensure the validity and reliability of the anemia estimates used in the analysis.

The dependent variable is “anemia,” which has been transformed into a binary variable coded as “1” if there is any kind of prevalence of anemia and “0” otherwise. The trends over the last two decades (1998–2021) and socioeconomic variations and heterogeneities based on socioeconomic factors have been examined along the dimensions of age, area of residence, caste, education, economic status, and different regions (which are considered as independent variables) among the Muslim women. To capture the prevalence of anemia and heterogeneities across various socioeconomic factors, the methods described below are used:

### 2.1 Cross-tabulation

This statistical method is used to analyze the percentage prevalence of anemia among Muslim women of reproductive age (15–49 years). Cross-tabulations allow this study to identify patterns and disparities in anemia prevalence by comparing proportions or percentages across these categories.

### 2.2 Concentration index

The Concentration Index is a widely used tool in health economics to measure inequality in health outcomes across different socioeconomic groups. It ranges from −1 to +1, where a negative value means the health condition (in this case, anemia) is more concentrated among the poorer segments of the population, and a positive value indicates a higher prevalence among the wealthier segments (48). A value of 0 implies perfect equality, that is, anemia is evenly distributed across all economic groups. This index is derived from a concentration curve, which plots the cumulative percentage of the health variable (e.g., anemia) against the cumulative percentage of the population, ranked by income or wealth. The farther the curve is from the diagonal line of equality, the greater the inequality (49). In this study, the CI is used to track how anemia prevalence among Muslim women varies with economic status across different NFHS rounds. It offers a summary statistic that not only captures the existence of disparity but also reveals its direction and magnitude, which is especially useful for policy targeting and equity monitoring (50).

### 2.3 Poor-rich (P/R) ratios

The P/R ratio is used to measure the disparity in anemia prevalence between women belonging to the poorest and richest wealth quintiles. It is defined as the ratio of the prevalence percentage of anemia in the poorest quintile to the prevalence percentage in the richest quintile.

A P/R ratio of 1 indicates no economic disparity, meaning anemia prevalence is equally distributed across the poorest and richest groups. A P/R ratio greater than 1 suggests higher anemia prevalence among women in the poorest wealth quintile compared to those in the richest quintile (50). This ratio is a simple and widely used measure to assess socioeconomic disparities in health and nutrition outcomes. It has been adopted in prior literature (e.g., Pathak and Singh, 2011) to summarize economic inequality, particularly in the context of undernutrition among children (48). The P/R ratio helps to highlight the disproportionate burden of anemia on economically disadvantaged populations.

### 2.4 Logistic regression model

The logistic regression model has been used by transforming the linear combination of predictor variables and coefficients into probabilities and odds; the relationship between socioeconomic factors, such as caste, age, place of residence, education, wealth quintiles, and region, and the probability of women experiencing anemia has been discerned. A logistic regression model was employed to assess the association between anemia prevalence and various socioeconomic and demographic factors, including caste, age, place of residence, education, wealth quintiles, and region. Odds ratios were estimated to determine the likelihood of anemia among Muslim women in each category, compared to relevant reference groups (51). In the context of this study, the odds ratio (OR) derived from the logistic regression model is used to determine the likelihood (or odds) of anemia among Muslim women of reproductive age in India based on various socioeconomic and demographic factors.

### 2.5 Spatial analysis

Across different states, the geographical variance using ArcGIS software is used to assess anemia prevalence among Muslim women, both at the state and regional levels. This approach highlights the importance of considering state and regional economic disparities to identify localized health challenges and tailor public health interventions to address the specific needs of different regions effectively.

## 3 Results and preliminary findings

### 3.1 Distribution of socioeconomic characteristics

**Figure 1:**
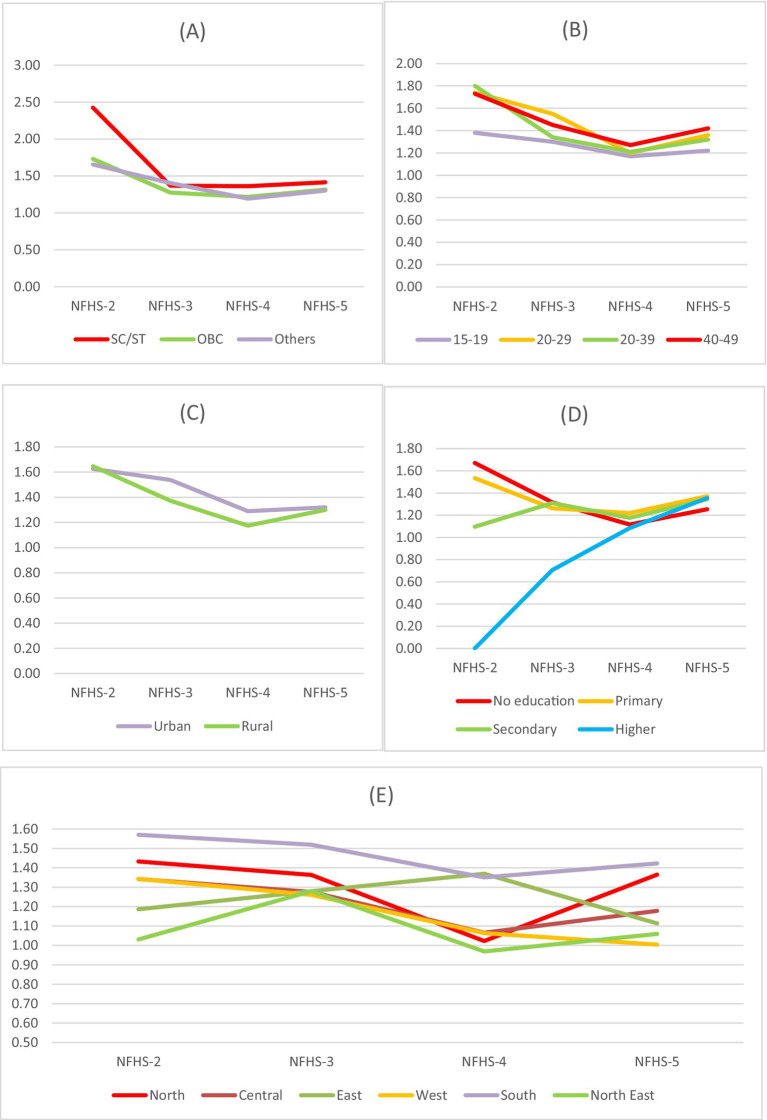
Trends in economic disparities with respect to anemia among Muslim women using P/R ratio across **(A)** caste, **(B)** age groups (in years), **(C)** residence, **(D)** educational groups, and **(E)** regions for NFHS-2 (1998-99), NFHS-3 (2005-06), NFHS-4 (2015-16) and NFHS-5 (2019-21).

Figure 1 provides a percentage breakdown of Indian women aged 15–49 years, categorized by various socioeconomic factors. These include religion (such as Muslim, Hindu, Christian, Sikh, Buddhist, Jain, and others), age (8, 15–48), caste (grouped as SC/ST, OBC, and Others), and place of residence (urban or rural). It also captures levels of education, ranging from no formal education to completed primary (5 years), secondary (10 years), and higher secondary (12 years). Additionally, the table organizes the data into wealth quintiles and different geographic regions, offering insight into the socioeconomic landscape of this population. This detailed categorization helps highlight the diverse socioeconomic background of women of reproductive age, which could be critical for examining health outcomes like anemia prevalence across different segments of the population. The data reveal a clear trend toward urbanization and increased education among women in India over the two decades, which likely contributes to better access to healthcare and nutritional resources. As more women move to urban areas and attain higher levels of education, awareness about health and nutrition improves, which could help reduce the prevalence of anemia. However, significant economic disparities persist, particularly between the wealthiest and poorest groups, which can limit access to essential resources like nutritious food and healthcare, especially for women in lower-income households. Regional differences also play a role, with certain areas experiencing population shifts that may influence healthcare distribution. Additionally, while the inclusion of more SC/ST and OBC women in surveys shows improved representation, these groups still face systemic challenges that could make them more vulnerable to anemia and other health issues. Overall, while some progress has been made, socioeconomic and regional inequalities continue to present significant challenges in addressing anemia among women in India (Table 1).

**Table 1:** Percentage distribution of socioeconomic characteristics of women in India (aged 15–49), based on NFHS (1998–2021).

Socioeconomic characteristics	NFHS-2	NFHS-3	NFHS-4	NFHS-5
Religion				
Hindu	81.75	80.52	80.57	81.36
Muslim	12.53	13.62	13.79	13.48
Christian	2.53	2.45	2.38	2.35
Sikh	1.59	1.79	1.66	1.57
Buddhist	0.76	0.81	0.92	0.63
Jain	0.37	0.33	0.18	0.23
Others	0.46	0.49	0.5	0.38
Age				
15–19	9.39	20.34	17.70	17.27
20–29	39.18	35.41	34.66	33.35
20–39	29.96	25.51	25.43	25.83
40–49	21.47	18.74	22.21	23.55
Caste				
SC/ST	27	26.73	29.55	31.18
OBC	32.91	39.30	43.42	42.92
Others	40.09	33.98	27.02	25.91
Place of residence				
Urban	26.18	32.82	34.62	32.49
Rural	73.82	67.18	65.38	67.51
Education				
No education	53.44	40.59	27.46	22.43
Primary	16.9	14.7	12.47	11.73
Secondary	21.77	37.41	47.31	50.18
Higher	7.89	7.3	12.76	15.65
Wealth quintiles				
Poorest	19.66	17.46	17.73	18.5
Poorer	19.99	18.99	19.57	20.00
Middle	20.15	20.17	20.55	20.52
Richer	20.01	20.99	21.15	20.81
Richest	20.18	22.4	21	20.17
Region				
North	12.87	13.28	13.59	14.11
Central	22.42	23.26	23.65	24.89
East	21.99	22.44	22.11	22.76
West	14.64	14.83	14.37	14.09
South	24.59	22.24	22.76	20.45
North East	3.49	3.95	3.52	3.69

SC/ST, Scheduled castes and scheduled tribes; OBC, Other backward castes. Author’s calculation based on NFHS-2 (1998–99), NFHS-3 (2005–06), NFHS-4 (2015–16) and NFHS-5 (2019–21) data.

### 3.2 Trends in anemia among Muslim women in India

We find evolving trends in anemia prevalence among Muslim women over time, as shown in Table 2. Anemia prevalence declined across most socioeconomic groups between NFHS-3 and NFHS-4, but rose again in NFHS-5. Regional disparities persist, with higher rates in the East and Northeast, lower in the West and Central regions, and the lowest in the South, reflecting potential regional influences (19). These findings highlight the shifting nature of anemia prevalence among Muslim women and underscore the need for continuous monitoring and targeted interventions, particularly in high-prevalence regions.

**Table 2:** Percentage prevalence of anemia among Muslim women in India (aged 15–49), by socioeconomic characteristics, based on NFHS (1998 -2021).

Socioeconomic characteristics	NFHS-2		NFHS-3		NFHS-4		NFHS-5	
	Any	Severe	Any	Severe	Any	Severe	Any	Severe
All-India	48.77	1.32	54.66	1.15	50.60	0.90	55.60	0.77
Caste								
SC/ST	54.25	1.71	53.90	2.26	51.87	0.81	56.49	1.16
OBC	46.98	1.93	52.69	1.16	50.49	1.02	52.05	0.72
Others	49.12	1.11	55.73	1.10	50.60	0.80	58.62	0.78
Age								
15–19	46.97	0.49	55.20	1.07	51.82	0.70	58.33	0.77
20–29	49.02	1.41	55.42	1.27	50.33	0.87	55.14	0.62
20–39	49.01	1.12	53.14	1.01	50.95	0.93	54.09	0.85
40–49	48.61	1.81	54.54	1.14	50.05	1.12	55.69	0.95
Place of residence								
Urban	42.41	1.33	50.37	1.11	48.86	0.87	50.82	0.73
Rural	52.14	1.32	57.27	1.18	52.05	0.93	58.83	0.80
Education								
No education	54.48	1.50	58.46	1.34	53.77	1.28	58.32	0.87
Primary	48.33	1.42	54.59	1.39	51.65	0.92	55.79	1.08
Secondary	37.87	1.02	50.42	0.80	48.86	0.69	54.97	0.67
Higher	32.67	0.00	41.71	0.74	45.66	0.61	51.15	0.60
Wealth quintiles								
Poorest	64.35	1.90	63.81	1.01	55.63	1.08	62.36	0.89
Poorer	56.42	1.55	60.12	1.14	52.41	0.88	60.30	0.80
Middle	49.21	1.48	55.03	1.42	51.47	1.04	56.31	0.93
Richer	41.67	1.39	50.98	1.31	48.69	0.90	52.26	0.76
Richest	37.94	0.44	44.76	0.79	45.92	0.63	46.65	0.48
Region								
North	47.51	1.45	52.51	2.41	50.11	1.27	57.79	1.10
Central	47.83	1.23	50.77	1.05	51.82	1.24	50.51	0.68
East	60.50	1.66	63.52	0.65	55.95	0.57	64.67	0.58
West	37.60	0.69	47.71	0.98	48.95	0.71	51.76	1.09
South	34.60	1.46	49.26	1.29	45.20	0.99	44.77	0.82
North East	72.12	0.40	57.58	1.58	41.41	0.40	59.15	0.69

SC/ST, scheduled castes and scheduled tribes; OBC, other backward castes. Author’s calculation based NFHS-2 (1998–99), NFHS-3 (2005–06), NFHS-4 (2015–16) and NFHS-5 (2019–21) data.

Studies show that Muslims consistently have higher anemia rates than Hindus, Christians, and other religious groups (52). Contributing factors include poverty, inadequate access to clean water, healthcare, and sanitation (39, 53). The Sachar Committee Report identifies additional socioeconomic disadvantages, including limited education, restricted social security and job access, and discrimination, particularly affecting Muslim women (54). Class and gender further shape anemia prevalence in India. Among Muslim women, anemia rose from 48.77% in NFHS-2 to 55.56% in NFHS-5, with a sharp increase between NFHS-4 and NFHS-5. Hindu women also experienced a rise, but the sharper trend among Muslims suggests a need for focused interventions. Education and wealth significantly influence anemia prevalence, with the highest rates among the uneducated and poorest women. These findings stress the importance of policies addressing economic and educational disparities to reduce anemia rates effectively.

### 3.3 Trends and differentials in anemia among Muslim women using the concentration index

Across all socioeconomic factors (Caste, Age, Residence, Education, and Region), the Concentration Index (CI) values are negative across all NFHS rounds (NFHS-2 to NFHS-5). Negative CI values indicate that anemia is more concentrated among poorer Muslim women across all survey rounds. SC/ST women consistently exhibit the highest negative CI values, meaning they suffer from the most significant economic disparity in anemia prevalence. In NFHS-2 (−0.157) vs. NFHS-5 (−0.057), the CI value has decreased significantly across castes, indicating a reduction in economic inequality over time. Younger women (15–19 years) have the least negative CI values, implying they experience the least socioeconomic inequality in anemia prevalence. Whereas women aged 20–39 and 40–49 consistently have higher negative CI values, indicating that older women in lower economic groups are more affected by anemia. The difference between urban (−0.045) and rural (−0.035) CI in NFHS-5 suggests that rural poor Muslim women remain more vulnerable to anemia. Women with no education have higher absolute CI values (−0.081 in NFHS-2 to −0.035 in NFHS-5), showing persistent inequality. The negative CI value for higher education (−0.054 in NFHS-5) suggests that even well-educated but economically disadvantaged women still face anemia-related inequalities, though less severe than before. The South region initially showed high inequality in NFHS-3 (−0.067) but later improved in NFHS-4 (−0.032) before slightly worsening in NFHS-5 (−0.046), indicating fluctuating progress. The West region displayed a steady reduction in inequality, reaching NFHS-5 (−0.006), while the East region experienced moderate fluctuations, with inequality rising slightly in NFHS-4 (−0.037) before significantly improving in NFHS-5 (−0.011). Central India showed an improving trend until NFHS-4 (−0.011) but saw a reversal in NFHS-5 (−0.030), suggesting a loss of earlier progress. The North region followed a similar pattern, with inequality improving in NFHS-4 (−0.017) but worsening again in NFHS-5 (−0.054). The North-East region maintained relatively low disparities overall, though inequality peaked in NFHS-3 (−0.036) before improving in NFHS-5 (−0.007) (Table 3).

**Table 3:** Trends in economic disparities in the prevalence of any anemia among Muslim women, based on the Concentration Index from NFHS (1998 -2021).

Socioeconomic characteristics	NFHS-2		NFHS-3		NFHS-4		NFHS-5	
	CI	SE	CI	SE	CI	SE	CI	SE
Caste	−0.107***	0.006	−0.067***	0.004	−0.035***	0.002	−0.056***	0.002
SC/ST	−0.157***	0.03	−0.032	0.021	−0.046***	0.006	−0.057***	0.006
OBC	−0.106***	0.013	−0.065***	0.008	−0.037***	0.003	−0.052***	0.003
Others	−0.104***	0.007	−0.068***	0.005	−0.032***	0.003	−0.049***	0.002
Age	−0.109***	0.006	−0.067***	0.004	−0.035***	0.002	−0.055***	0.002
15–19	−0.073***	0.021	−0.048***	0.009	−0.023***	0.004	−0.034***	0.004
20–29	−0.112***	0.009	−0.084***	0.007	−0.036***	0.003	−0.057***	0.003
20–39	−0.117***	0.011	−0.056***	0.009	−0.035***	0.004	−0.057***	0.004
40–49	−0.111***	0.014	−0.072***	0.011	−0.044***	0.004	−0.068***	0.004
Place of residence	−0.107***	0.006	−0.067***	0.004	−0.035***	0.002	−0.056***	0.002
Urban	−0.067***	0.01	−0.054***	0.006	−0.035***	0.003	−0.045***	0.003
Rural	−0.099***	0.007	−0.057***	0.006	−0.024***	0.002	−0.035***	0.002
Education	−0.107***	0.006	−0.067***	0.004	−0.035***	0.002	−0.056***	0.002
No education	−0.081***	0.007	−0.048***	0.006	−0.021***	0.003	−0.035***	0.003
Primary	−0.081***	0.014	−0.051***	0.011	−0.026***	0.005	−0.058***	0.005
Secondary	−0.031*	0.016	−0.054***	0.007	−0.028***	0.003	−0.059***	0.002
Higher	−0.014	0.03	−0.04**	0.018	−0.022***	0.007	−0.054***	0.006
Region	−0.107***	0.006	−0.067***	0.004	−0.035***	0.002	−0.056***	0.002
North	−0.061***	0.012	−0.044***	0.009	−0.017***	0.004	−0.054***	0.003
Central	−0.04**	0.018	−0.037***	0.011	−0.011***	0.003	−0.03***	0.004
East	−0.051***	0.011	−0.032***	0.008	−0.037***	0.004	−0.011***	0.003
West	−0.04*	0.022	−0.035**	0.014	−0.013*	0.008	−0.006	0.006
South	−0.05***	0.018	−0.067***	0.01	−0.032***	0.006	−0.046***	0.006
North East	−0.002	0.011	−0.036***	0.011	−0.027***	0.006	−0.007*	0.004

SC/ST, Scheduled castes and Scheduled Tribes; OBC, Other Backward Classes; CI refers to the Concentration Index, which measures the socioeconomic inequality in the prevalence of anemia among women; SE, standard error. *p < 0.1, **p < 0.05, ***p < 0.01. Author’s calculation based on NFHS-2 (1998–99), NFHS-3 (2005–06), NFHS-4 (2015–16) and NFHS-5 (2019–21) data using STATA.

### 3.4 Trends and differentials in anemia among Muslim women using the P/R ratio

The P/R ratio declined from 2.43 (NFHS-2) to 1.37 (NFHS-3), indicating reduced caste-based inequality, but rose slightly to 1.41 in NFHS-5, signaling widening disparities. Women aged 40–49 years had the highest P/R ratio in NFHS-5 (1.42), while adolescents (15–19 years) consistently had the lowest, declining from 1.38 (NFHS-2) to 1.22 (NFHS-5), suggesting lower disparities among young women. Urban areas showed higher P/R ratios than rural areas in all rounds except NFHS-5. Urban disparities declined from 1.63 (NFHS-2) to 1.29 (NFHS-4) but slightly increased to 1.32 (NFHS-5). Rural disparities followed a similar pattern, dropping from 1.65 (NFHS-2) to 1.17 (NFHS-4) before rising to 1.30 (NFHS-5). Women with no education had the highest disparities in NFHS-2 (1.67) and NFHS-3 (1.32), which declined to 1.12 (NFHS-4) but rose again to 1.25 (NFHS-5). Highly educated women had the lowest ratio in NFHS-3 (0.71), but it increased to 1.36 in NFHS-5, indicating growing inequality among them. Primary and secondary education groups also saw rising disparities, reaching 1.37 and 1.34 in NFHS-5. Regional trends were mixed: the East region saw a peak in NFHS-4 (1.37) before dropping to 1.11 (NFHS-5), reflecting reduced inequality, while the North showed worsening disparities, rising from 1.02 (NFHS-4) to 1.37 (NFHS-5). Overall, the decline in inequality from NFHS-2 to NFHS-4 suggests improved equity in anemia prevalence, but NFHS-5 shows a reversal, with rising disparities across most categories. SC/ST women, older women (40–49 years), rural residents, uneducated women, and those in the South, Central, and North regions face consistently higher disparities, underscoring the need for targeted interventions to address anemia among Muslim women in India.

### 3.5 Trends and differentials in anemia among Muslim women using odds ratios

Table 4 presents Odds Ratios (OR) for anemia prevalence among Muslim women across socioeconomic and demographic factors from NFHS-2 to NFHS-5, offering insights into the likelihood of anemia based on different characteristics. OR values above 1 indicate an increased likelihood of anemia compared to the reference group, while values below 1 suggest reduced odds.

**Table 4:** Trends and differentials in the prevalence of any anemia among Muslim women, based on odds ratios from NFHS (1998–2021).

Socioeconomic characteristics	NFHS-2		NFHS-3		NFHS-4		NFHS-5	
	Odds ratio	[95% CI]	Odds ratio	[95% CI]	Odds ratio	[95% CI]	Odds ratio	[95% CI]
Caste (SC/ST^®^)								
OBC	0.934	[0.718, 1.213]	1.316***	[1.105, 1.568]	−0.031	[−0.084, 0.022]	0.914***	[0.865, 0.965]
Others	0.874	[0.681, 1.122]	1.361***	[1.151, 1.609]	−0.019	[−0.071, 0.034]	1.043	[0.987, 1.101]
Age (15–19)^®^								
20–29	1.138	[0.971, 1.334]	0.98	[0.897, 1.069]	−0.037**	[−0.074, 0]	0.852***	[0.818, 0.887]
20–39	1.123	[0.952, 1.324]	0.948	[0.859, 1.046]	−0.057***	[−0.099, −0.016]	0.812***	[0.776, 0.849]
40–49	1.084	[0.91, 1.292]	0.922	[0.824, 1.033]	−0.091***	[−0.137, −0.045]	0.858***	[0.817, 0.902]
Residence (Urban^®^)								
Rural	0.903*	[0.808, 1.009]	0.956	[0.884, 1.034]	0.078***	[0.046, 0.109]	1.199***	[1.158, 1.242]
Education (No education^®^)								
Primary	0.956	[0.848, 1.078]	0.897**	[0.81, 0.994]	−0.077***	[−0.12, −0.033]	0.933***	[0.888, 0.979]
Secondary	0.8***	[0.702, 0.913]	0.907**	[0.828, 0.993]	−0.109***	[−0.145, −0.073]	0.968	[0.93, 1.007]
Higher	0.716***	[0.565, 0.907]	0.794**	[0.667, 0.946]	−0.167***	[−0.227, −0.107]	1.015	[0.956, 1.077]
Wealth (Poorest^®^)								
Poorer	0.737***	[0.624, 0.871]	0.863**	[0.752, 0.99]	−0.039*	[−0.084, 0.006]	0.946**	[0.904, 0.99]
Middle	0.719***	[0.608, 0.849]	0.697***	[0.61, 0.797]	−0.062**	[−0.11, −0.015]	0.893***	[0.85, 0.937]
Richer	0.594***	[0.5, 0.706]	0.62***	[0.539, 0.713]	−0.072***	[−0.123, −0.021]	0.847***	[0.804, 0.893]
Richest	0.499***	[0.407, 0.611]	0.526***	[0.45, 0.614]	−0.131***	[−0.189, −0.073]	0.704***	[0.663, 0.747]
Regions (North^®^)								
Central	1.01	[0.87, 1.172]	0.85***	[0.762, 0.948]	−0.002	[−0.04, 0.036]	0.571***	[0.547, 0.597]
East	1.54***	[1.335, 1.776]	1.244***	[1.113, 1.39]	0.106***	[0.062, 0.149]	0.823***	[0.786, 0.863]
West	0.857*	[0.727, 1.01]	1.055	[0.929, 1.199]	−0.115***	[−0.177, −0.053]	0.656***	[0.62, 0.695]
South	0.67***	[0.584, 0.769]	1.017	[0.914, 1.132]	−0.232***	[−0.281, −0.183]	0.478***	[0.455, 0.502]
North East	2.119***	[1.794, 2.503]	0.746***	[0.66, 0.845]	−0.513***	[−0.562, −0.463]	0.623***	[0.593, 0.656]
_cons	1.506**	[1.074, 2.111]	1.327**	[1.066, 1.65]	0.243***	[0.168, 0.318]	2.272***	[2.095, 2.463]

SC/ST, Scheduled castes and Scheduled Tribes; OBC, Other Backward Classes; 95% CI, confidence interval at 95% level of significance. Author’s calculation based on NFHS-2 (1998–99), NFHS-3 (2005–06), NFHS-4 (2015–16) and NFHS-5 (2019–21) data using STATA; cons represents the intercept, i.e., the baseline odds of anemia for the reference categories of all covariates. *p* <0.1 = *, <0.05 = **, <0.01 = ***.

Analyzing by caste, OBC Muslim women had higher odds of anemia in NFHS-3 (OR = 1.316, *p* < 0.001), but these odds dropped in subsequent rounds, reaching 0.944 in NFHS-5, suggesting diminishing caste-based disparities. Similarly, Other caste Muslim women showed elevated odds in NFHS-3 (OR = 1.361, p < 0.001), but these differences also became non-significant over time, indicating a reduction in caste-related anemia disparity. Regarding age, women in the 20–29 and 30–39 age groups had slightly increased odds in early rounds but lower odds in NFHS-4 and NFHS-5. For instance, in NFHS-5, the 20–29 group had an OR of 0.852 (*p* < 0.001), suggesting younger women (15–19) are more at risk. The 40–49 group shows a similar decrease in odds over time, with anemia risks diminishing as women age, particularly in recent rounds.

Residence impacts anemia likelihood, with rural women showing lower but non-significant odds in NFHS-2 (OR = 0.903). However, by NFHS-5, rural Muslim women exhibited higher odds of anemia (OR = 1.199, *p* < 0.001), marking an increasing rural–urban disparity, where rural women now face higher anemia risks. Education consistently emerges as a protective factor. Women with higher education showed reduced odds across rounds, although the effect was not statistically significant in NFHS-5 (OR = 1.015). Primary and secondary education also lowered anemia odds in NFHS-4 and NFHS-5, with secondary education yielding significantly reduced odds in NFHS-5 (OR = 0.933, p < 0.001), confirming the protective influence of educational attainment.

In terms of wealth, there is a clear gradient in anemia odds across quintiles, with wealthier Muslim women consistently displaying lower odds. In NFHS-5, those in the richest quintile had an OR of 0.841 (*p* < 0.001), indicating significantly reduced anemia likelihood compared to the poorest. Even those in intermediate wealth quintiles showed reduced odds in later rounds; for example, the second quintile in NFHS-5 had an OR of 0.946 (*p* < 0.01), underscoring the beneficial impact of economic stability. Regional disparities are also pronounced, as women in the East and Northeast consistently showed higher anemia odds compared to the North. In NFHS-5, the East region had an OR of 1.214 (*p* < 0.01), while the Northeast showed lower odds (OR = 0.623, *p* < 0.01). Women in the South and West generally exhibited lower anemia odds, with the South achieving significantly lower odds in NFHS-5 (OR = 0.442, *p* < 0.001), potentially reflecting improved health and nutritional resources in these regions.

Across all three NFHS rounds, there is a significant difference in the odds of anemia based on caste. SC/ST populations consistently have the highest odds of anemia, followed by OBC and Others. Notably, in NFHS-5, there is a significant decrease in the odds of anemia among all caste groups compared to NFHS-3 and NFHS-4. Urban areas consistently show lower odds of anemia compared to rural areas in all three NFHS rounds. While urban areas showed a decrease in the odds of anemia from NFHS-3 to NFHS-4, it increased slightly in NFHS-5. Women with higher levels of education tend to have lower odds of anemia. The gap in anemia prevalence between education levels is most pronounced in NFHS-3 and narrower in NFHS-4 and NFHS-5. Even in NFHS-5, women with no education or primary education still exhibit higher odds of anemia compared to those with secondary or higher education. There is a clear gradient in anemia prevalence across wealth quintiles, with the poorest quintile consistently having the highest odds of anemia. In all NFHS rounds, the richest quintile has the lowest odds of anemia. Notably, there is a significant decrease in anemia prevalence among the poorest quintile in NFHS-5 compared to NFHS-3 and NFHS-4. Significant regional disparities exist in the odds of anemia across all three NFHS rounds. In NFHS-3, the North and East regions had the highest anemia prevalence, while the South region had the lowest. In NFHS-4, the Central region had the highest anemia prevalence, and the Northeast region had the lowest. In NFHS-5, the North and Central regions again show higher anemia prevalence, while the South and Northeast regions exhibit lower prevalence. Notably, in NFHS-5, there is a significant decrease in anemia prevalence in the North region compared to NFHS-4. These findings highlight the persistence of socioeconomic disparities in anemia prevalence among Muslim women in India, with caste, education, wealth, place of residence, and region all playing significant roles. While there have been improvements in certain areas (e.g., decreased anemia prevalence among the poorest quintile in NFHS-5), ongoing efforts are needed to address and further reduce anemia rates, especially among vulnerable populations.

## 4 Discussion

**Figure 2:**
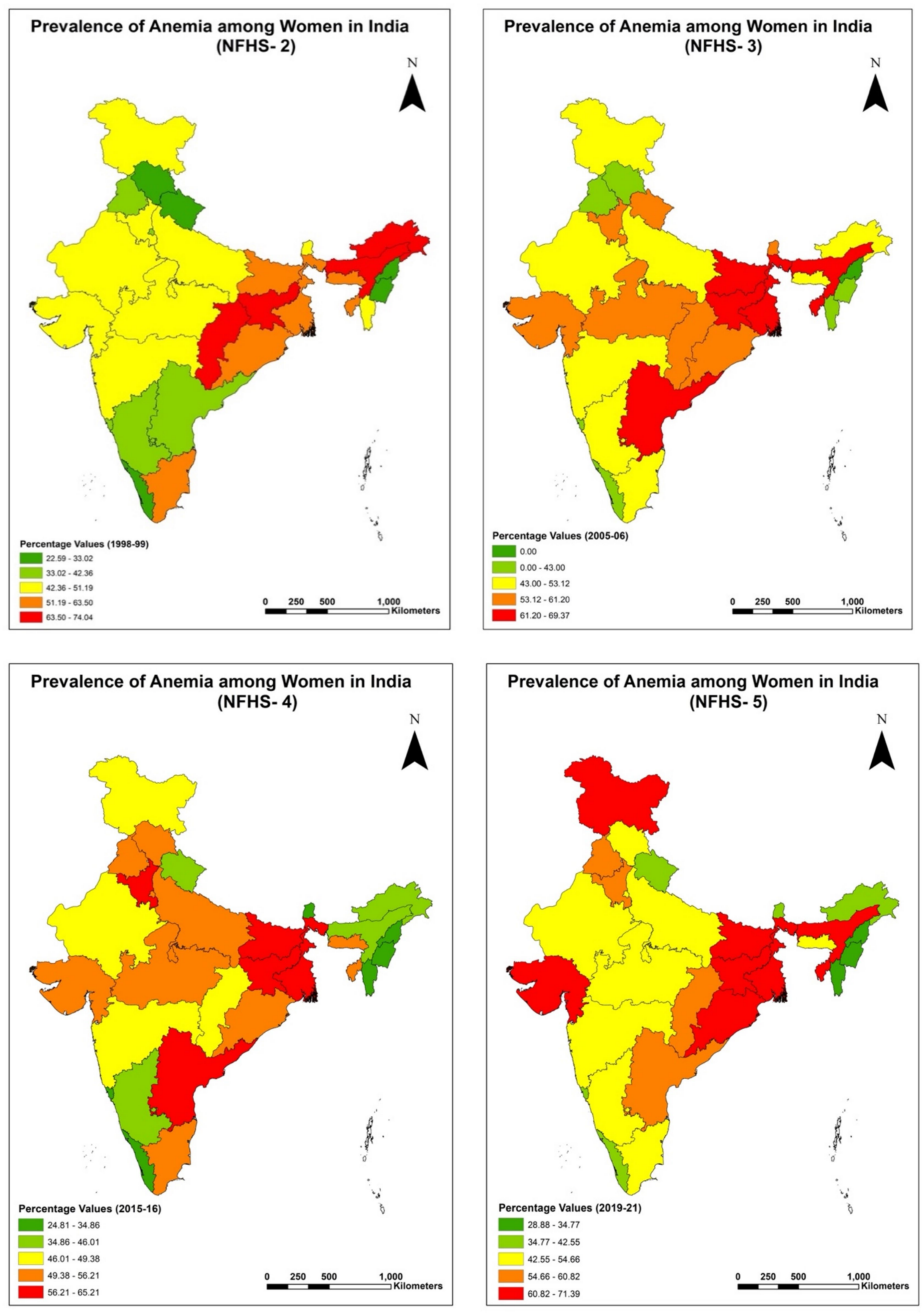
Regional analysis of percentage prevalence of any anemia among overall women (15–49 years) in India for NFHS-2 (1998–99), NFHS-3 (2005–06), NFHS-4 (2015–16) and NFHS-5 (2019–21).

While the overall analysis shows that anemia prevalence among women in India has increased over the two decades, a closer look through the socioeconomic lens tells the tale of disparities. The present study highlights the significant burden of anemia among Muslim women in India, emphasizing the influence of socioeconomic, demographic, and geographical factors. Using data from NFHS-2 to NFHS-5, the study demonstrates the unequal prevalence of anemia among Muslim women across different classes, age groups, caste affiliations, and wealth quintiles. The North and Central regions consistently exhibit higher anemia prevalence, while the South and Northeast regions generally display lower rates. The East region emerges as an exception, demonstrating the lowest prevalence of anemia in most categories. Figure 1 presents a state-wise choropleth map of anemia prevalence among all women across NFHS rounds, highlighting consistently high burden in several eastern and central states. Figure 2 focuses specifically on Muslim women, revealing similar spatial patterns, though with sharper disparities in states like Bihar, Assam, and Uttar Pradesh. These maps visually reinforce the statistical findings on regional heterogeneity and emphasize the need for geographically targeted policy responses. The regional variations underscore the influence of geographical factors on anemia prevalence and emphasize the need for tailored interventions addressing region-specific challenges. Recent regional studies across Indian states further validate the disparities identified in our national-level analysis and enhance the external validity of our findings. For instance, Mog and Ghosh conducted a spatial–temporal assessment in Maharashtra and highlighted persistent anemia among women of reproductive age, especially in under-resourced districts (40). In Bihar, Chauhan et al. found a high prevalence of anemia among adolescents, attributing it to limited dietary diversity and poor reproductive health education (55). Similarly, Banerjee and Duflo demonstrated that anemia among women in rural Bihar could be significantly reduced through iron-fortified salt interventions, underscoring the importance of targeted nutrition strategies (56). Biradar documented anemia prevalence among preconception young married women in India, with regional clusters of vulnerability across northern states (18). In West Bengal, Rao et al. found micronutrient deficiencies to be a leading cause of anemia among rural children, which likely extends into adolescence and adulthood (3). Bharati et al. analyzed anemia trends among reproductive-aged women and found widening rural–urban and state-level disparities over time (57). These findings align with our results and further illustrate the role of regional health infrastructure, social determinants, and nutrition behavior in shaping anemia outcomes. Additionally, Gupta et al. highlighted that women’s empowerment and intra-household decision-making power are closely linked to iron status, reinforcing the importance of gender-sensitive strategies across regions (8).

Furthermore, this research highlights the enduring impact of socioeconomic characteristics on anemia prevalence among Muslim women in India. Caste, education, wealth status, and place of residence all demonstrate significant associations with anemia risk. Scheduled Castes and Scheduled Tribes (SC/ST) consistently show higher anemia rates, necessitating targeted interventions to address disparities within these communities. Educational attainment and wealth status exhibit a clear gradient, with higher levels of education and economic prosperity associated with lower anemia prevalence. Urban areas generally fare better than rural areas, though a slight increase in anemia prevalence in urban areas in the most recent NFHS-5 round warrants attention. The findings also reveal that marginalized groups, particularly Scheduled Tribes (ST) and Scheduled Castes (SC), face a disproportionately higher risk of anemia compared to other social groups. This trend is consistent with prior research indicating the socioeconomic disadvantages faced by these communities (3, 14, 41). ST and SC Muslim women, especially those from economically disadvantaged households, exhibit the highest prevalence of anemia (24). These groups often face challenges such as poverty, poor access to healthcare, limited educational opportunities, and inadequate nutrition, which exacerbate their vulnerability to anemia (24). Previous studies have also observed a similar trend, with ST and SC populations experiencing higher rates of malnutrition, morbidity, and anemia due to restricted access to food, healthcare, and education (16, 40, 58). Additionally, the lack of political representation and slower poverty alleviation efforts have further marginalized the ST population compared to SC groups, leading to poorer health outcomes (8). The findings also highlight that rural Muslim women, particularly those from ST and SC communities, are more susceptible to anemia. Rural areas often lack basic infrastructure, including healthcare services, clean water, and sanitation facilities, which increases the prevalence of anemia (59). Studies by Biradar and Sharif et al. align with these results, showing that women in rural areas, especially those belonging to disadvantaged groups, have consistently exhibited higher anemia prevalence across 1998–2021 (18, 24). Economic status and education emerge as critical determinants of anemia prevalence among Muslim women. Women from poorer households are more likely to suffer from anemia, as economic deprivation limits their access to nutritious food and healthcare. Mog et al. found similar associations between economic class and anemia prevalence, emphasizing the role of household income in determining health outcomes (60). Education also plays a protective role, as higher levels of education are associated with better health awareness, improved dietary diversity, and greater access to healthcare services (16). Studies suggest that women’s empowerment within households is associated with better health outcomes, including reduced anemia prevalence (61). Autonomy in fertility decisions and delayed marriages further contribute to improved maternal health, as women in such households are less likely to experience frequent pregnancies or low birth weights (35).

The findings of this study align with the theory of intersectionality, which explains how multiple social identities, such as caste, class, gender, and religion, interact to create unique experiences of privilege and disadvantage (62). Muslim women in India face intersecting forms of discrimination that amplify their risk of anemia (63). For instance, a rural Muslim woman belonging to an ST or SC community is more likely to experience poverty, inadequate healthcare access, and limited educational opportunities, all of which contribute to higher anemia prevalence (42, 44). The Sachar Committee Report revealed that Muslim communities face systemic socioeconomic disadvantages, including lower levels of education, limited access to social security, and employment discrimination (43). These factors disproportionately affect Muslim women, further marginalizing their position in society and increasing their susceptibility to anemia (54). This intersection of caste, class, religion, and gender shapes the health outcomes of Muslim women, highlighting the need for targeted interventions that address these overlapping inequities. The results of this study are consistent with prior research on anemia in India. Studies have shown that caste identity significantly influences anemia prevalence, even after controlling for demographic factors such as education and household wealth (33, 64). Similarly, Other studies have observed higher anemia rates among socio-economically disadvantaged groups, particularly in rural areas (24, 34). These findings reinforce the idea that structural inequalities, manifested through caste, class, and gender, play a central role in shaping health outcomes in India.

Despite the implementation of major national initiatives such as Anemia Mukt Bharat, Poshan Abhiyaan, and the Iron and Folic Acid Supplementation Programme, our findings suggest that these efforts have not adequately reached all vulnerable groups. The persistently high anemia prevalence among Muslim women, particularly those in rural areas, SC/ST communities, and economically disadvantaged households, along with the recent increase in anemia among urban women in NFHS-5, indicates limited impact or uneven coverage of current interventions. These trends suggest that structural barriers, such as poor healthcare access, sociocultural exclusion, and low awareness, may be undermining the effectiveness of national programs. Ahmad et al. highlighted several bottlenecks in the public health supply chain for iron and folic acid supplementation in India, reinforcing the need to address logistical and systemic constraints under the Anemia Mukt Bharat strategy (65). As such, these “negative” findings are not just limitations, but they are crucial signals for policy reassessment and provide evidence-based justification for strengthening program implementation, ensuring intersectional targeting, and improving monitoring and accountability within existing health and nutrition frameworks.

**Figure 3:**
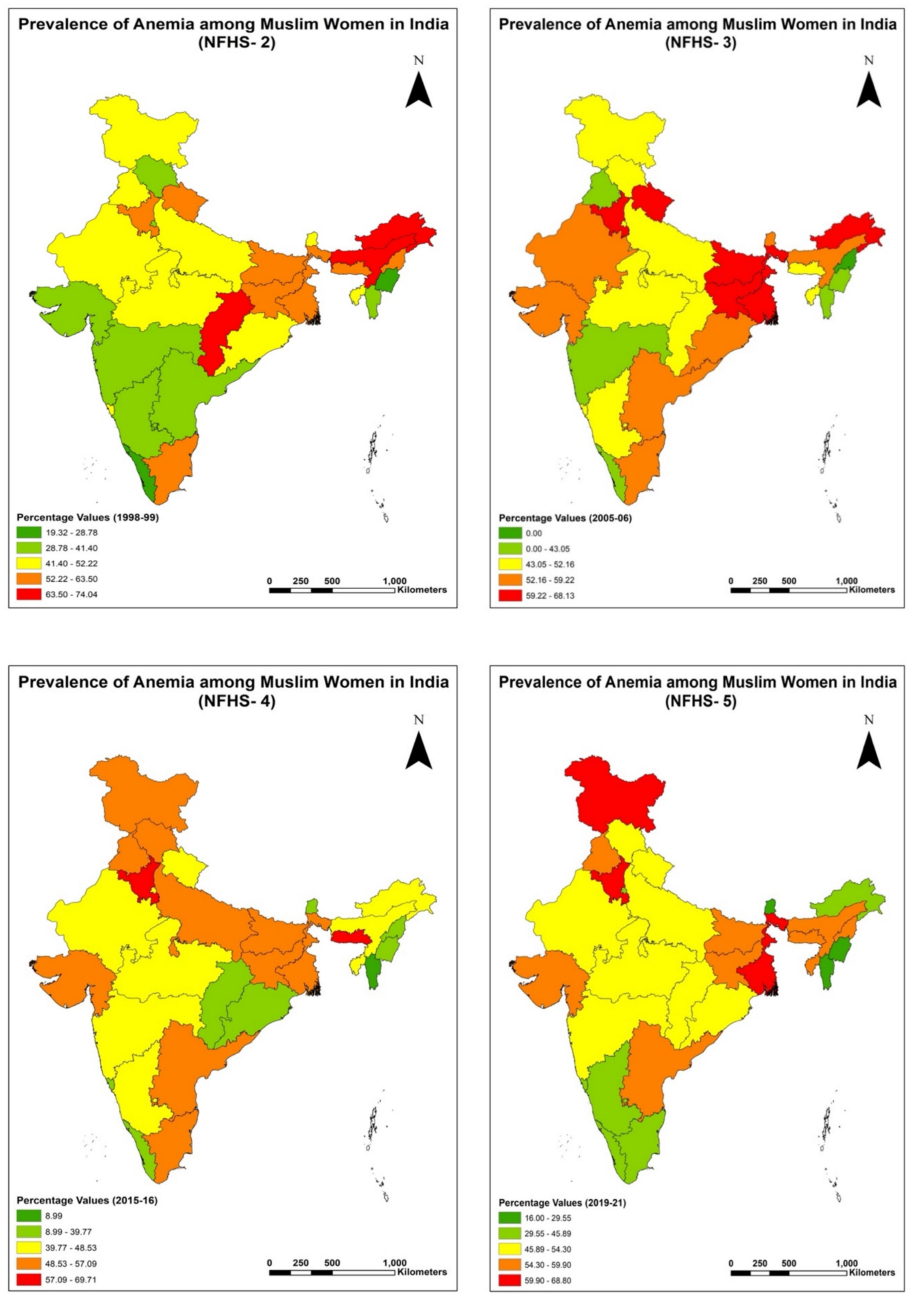
Regional analysis of Percentage prevalence of any anemia among Muslim women (15–49 years) in India for NFHS-2 (1998–99), NFHS-3 (2005–06), NFHS-4 (2015–16) and NFHS-5 (2019–21).

These findings have significant implications for public health policies aimed at reducing anemia prevalence in India. Addressing anemia among Muslim women requires a comprehensive approach that considers the intersections of caste, class, gender, and place of residence. Targeted interventions, such as providing iron and folic acid supplements, promoting dietary diversity, and improving access to healthcare services, are essential for reducing anemia prevalence among marginalized groups. Policymakers must also address the structural barriers that perpetuate health inequities. This includes improving access to education and employment opportunities for Muslim women, addressing discriminatory practices in healthcare delivery, and ensuring the availability of basic amenities such as clean water and sanitation. Strengthening the healthcare infrastructure in rural areas and prioritizing the needs of ST and SC populations can further reduce the disparities in the prevalence of anemia among women of reproductive age in India (Figure 3).

## 5 Limitations and future research

This study has certain limitations that should be considered when interpreting the findings. Being a cross-sectional study, it cannot establish cause-and-effect relationships between socioeconomic factors and anemia prevalence. The analysis relies on secondary data from the NFHS, which may not fully capture the contextual factors influencing anemia, such as cultural practices or dietary habits. Future research could explore these intersections among diverse population groups. Additionally, longitudinal studies are recommended to assess the long-term impacts of socioeconomic and demographic factors on anemia prevalence, providing a more comprehensive understanding of its determinants.

## 6 Conclusion

India has progressed rapidly in terms of economic growth in the past three decades, but still suffers from substantial socioeconomic (including religion and gender-based) inequalities in economic and non-economic (such as health) outcomes. Within health, hunger and malnutrition have been chronic problems and challenges in Indian society, thereby causing more serious problems like anemia. Although progress has been made in reducing anemia prevalence in some demographic groups, women from rural areas, Scheduled Castes (SC), and Scheduled Tribes (ST), within Muslim households, consistently exhibit disproportionately higher rates compared to their urban and higher-caste counterparts (40, 58). These disparities reveal the structural barriers to achieving health equity in India.

Our findings indicate a modest but meaningful decline in economic disparities in anemia prevalence among Muslim women in India across the NFHS rounds. This trend is reflected in the consistently negative Concentration Index (CI) values and the gradual decrease in their absolute magnitude over time, suggesting a pro-poor distribution of anemia and some progress toward reducing inequality. However, this progress has not been uniform. Persistent regional disparities point to the need for localized and context-specific public health strategies. These patterns have important implications for policymakers and health practitioners, reinforcing the urgency of sustained and targeted efforts to reduce anemia, particularly among socially and economically disadvantaged populations. Drawing on four rounds of NFHS data spanning over two decades, this study offers timely evidence on both the progress made and the enduring gaps in anemia outcomes among Muslim women of reproductive age in India. Nonetheless, the persistence of regional disparities in anemia prevalence remains a concern.

In light of these findings, it is imperative for policymakers and healthcare providers to prioritize sustained efforts aimed at promoting health equity and reducing the prevalence of anemia among economically disadvantaged populations, particularly among Muslim women. Strategies should encompass region-specific initiatives to address regional disparities effectively. Overall, this research contributes to the broader discourse on public health and underscores the importance of continued vigilance and targeted interventions in the pursuit of improved health outcomes and reduced socioeconomic disparities among women of reproductive age in India.

## Data Availability

Publicly available datasets were analyzed in this study. This data can be found at: https://www.dhsprogram.com/data/dataset_admin.
